# Colonic Adenocarcinoma Presenting as Abdominal Wall Necrosis: A Case Report

**DOI:** 10.7759/cureus.87895

**Published:** 2025-07-14

**Authors:** Ashlyn White, Adam D Tickal, Timothy Christopher

**Affiliations:** 1 General Surgery, Grandview Medical Center, Birmingham, USA; 2 Surgery, Edward Via College of Osteopathic Medicine - Auburn Campus, Auburn, USA

**Keywords:** abdominal wall necrotizing fasciitis, cecal adenocarcinomas, colorectal cancer, invasive colon cancer, peritoneal biopsy

## Abstract

While typical presentations of colorectal cancer include gastrointestinal symptoms such as changes in bowel habits, anemia, and hematochezia, it can also present atypically, including direct invasion of adjacent structures such as the abdominal wall. This report details a rare case of cecal adenocarcinoma manifesting as rapidly progressive abdominal wall necrosis in a 43-year-old woman, highlighting the diagnostic challenge in such unusual presentations. The case underscores the importance of maintaining a high index of suspicion for underlying malignancy in atypical clinical scenarios and illustrates the complexity of balancing wound care, infection control, and oncologic management to optimize patient outcomes.

## Introduction

Colorectal cancer is the fourth most common cancer and the second most common cause of cancer-related death in the US [1]. Colorectal cancers can present with constitutional symptoms such as fever, weight loss, and malaise; however, they can also present with more colorectal-specific symptoms such as iron deficiency anemia, hematochezia, as well as changes in bowel habits and stool caliber [2]. It is important to note that despite these well-known presentations, colorectal cancer may appear in rarer forms, such as direct invasion of nearby structures, and even penetration into the abdominal wall [3]. When the clinical scenario appears unusual, prompt investigation must ensue to facilitate appropriate diagnosis and treatment.

The aim of this article was to present an extraordinarily rare case of cecal adenocarcinoma presenting as rapidly progressive abdominal wall necrosis, and to emphasize the need to keep a high index of suspicion for underlying malignancy when the clinical picture is confounded by an atypical acute abdominal wall infection.

This article was previously presented as a poster at the American Society of Colon and Rectal Surgeons (ASCRS) Annual Scientific Meeting on May 10th, 2025.

## Case presentation

A 43-year-old previously healthy female patient presented to the emergency department with complaints of worsening of a lower abdominal wound and associated surrounding pain. She reported first noticing the wound approximately two days prior and had since developed purulent drainage with a foul smell. She denied any known weight loss, gastrointestinal bleeding, and changes in bowel movements. Her medical history was significant for hypertension and polycystic ovarian syndrome, and her surgical history was significant only for cosmetic abdominoplasty 15 years prior. She reported no previous colonoscopy and cited no known personal or family history of cancer. 

**Figure 1:**
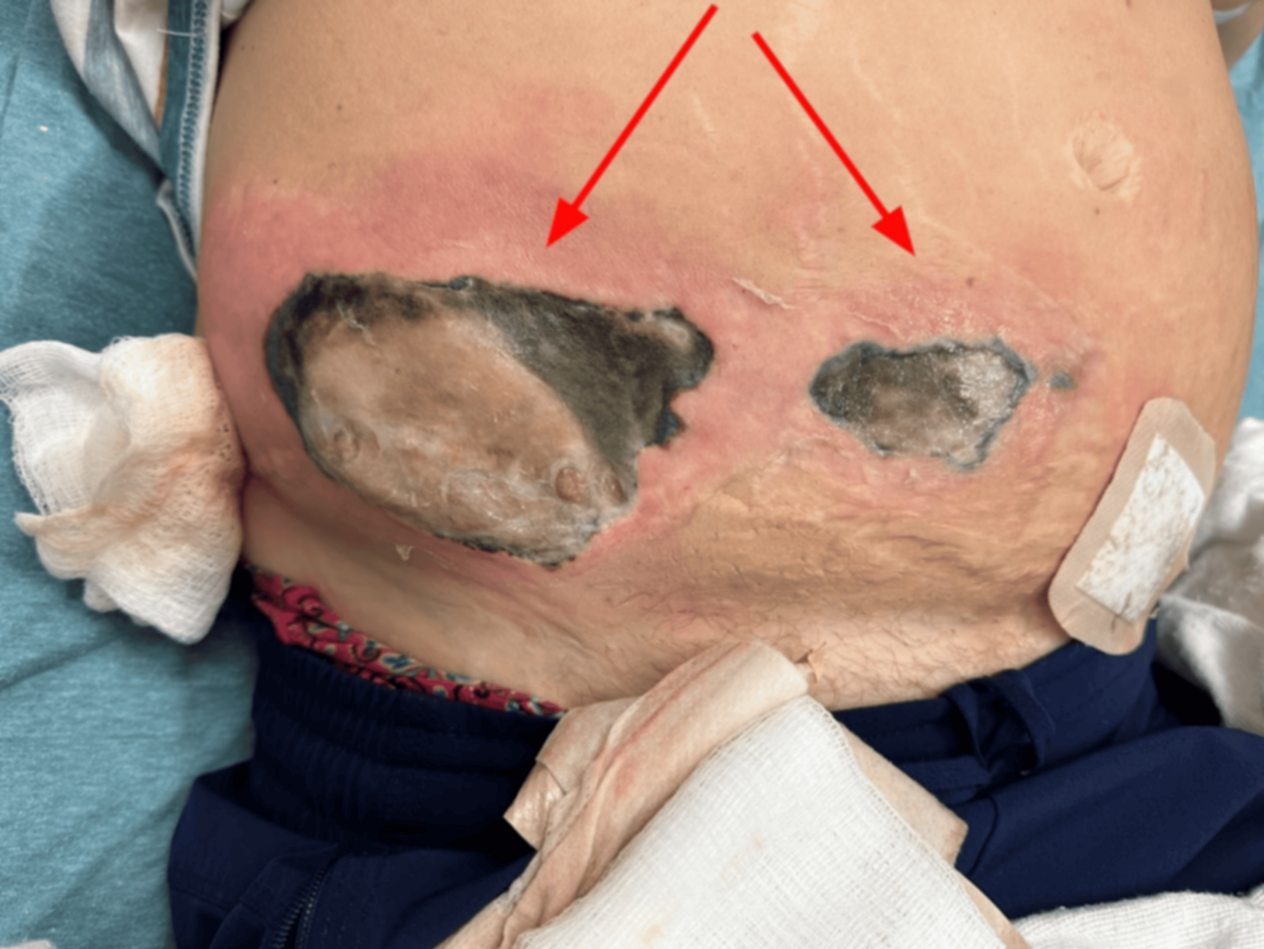
Anterior abdominal wounds with necrotic features and surrounding erythema.

On presentation, she was noted to be hypotensive and tachycardic. Her abdominal examination revealed two large, seemingly connected necrotic wounds to her lower abdomen with surrounding erythema, as seen in Figure 1. Laboratory data on admission demonstrated leukocytosis (white blood cells 18 μL (normal range: 4.0-10 μL)) and profound anemia (hemoglobin 4.2 g/dL (normal range: 13.5-17 g/dL), hematocrit 16.8% (normal range: 39-50%)). Tumor markers were notable for carcinoembryonic antigen (CEA) 11.7 ng/ml (normal range: 0-3.0ng/mL). 

**Figure 2:**
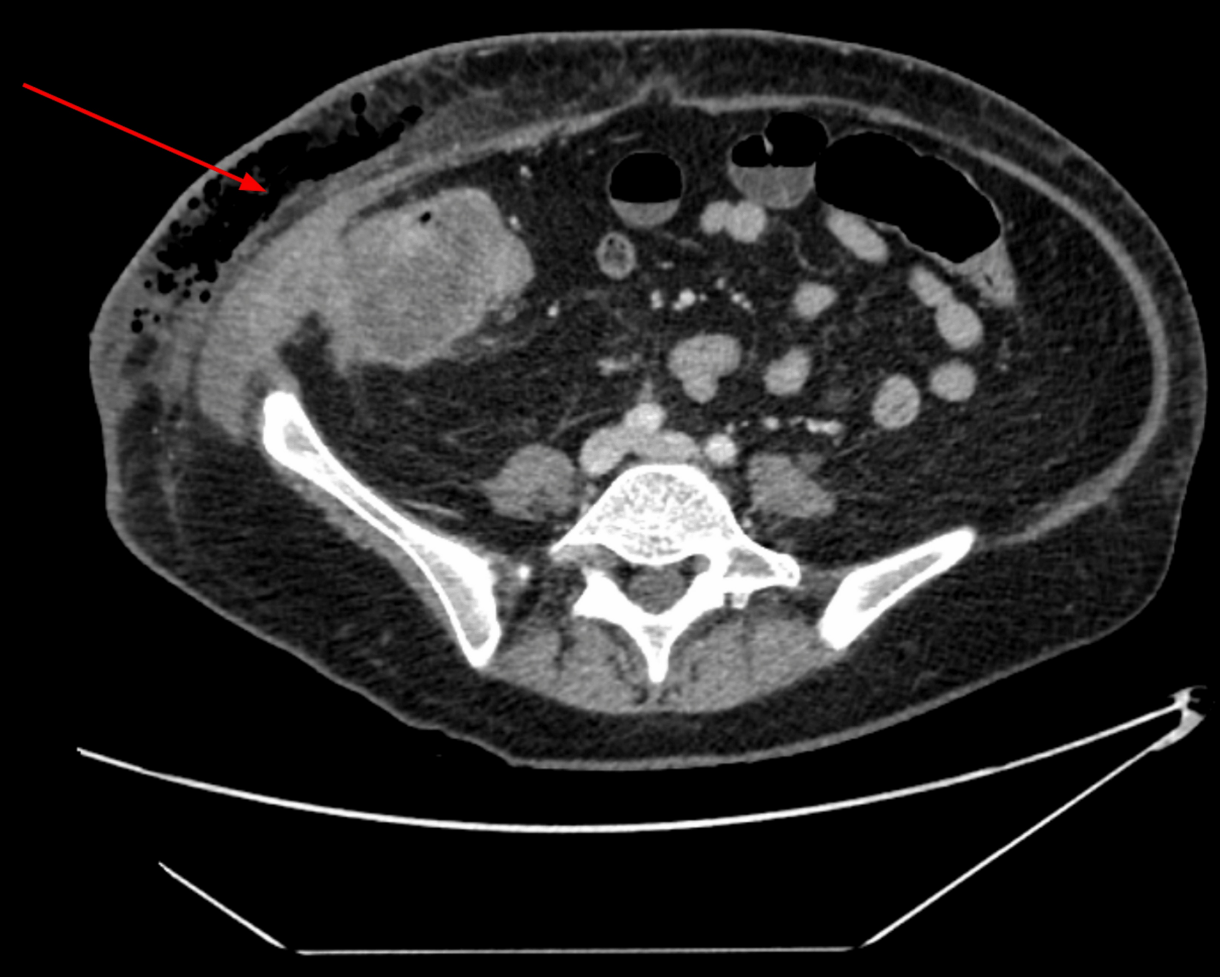
CT scan with IV contrast: axial image showing an inflamed colon with masslike thickening of the cecum concerning for primary colonic malignancy contiguous with the abdominal wall fluid collection and subcutaneous emphysema.

Initial imaging with computed tomography (CT) of the chest, abdomen, and pelvis with IV contrast identified extensive prominent subcutaneous emphysema in the right lower quadrant with abdominal wall fluid collections concerning for necrotizing fasciitis. An inflamed colon with mass-like thickening of the cecum concerning for primary colonic malignancy can be seen contiguous with the abdominal wall disease, as seen in Figure 2. No evidence of distant metastasis was identified.

**Figure 3:**
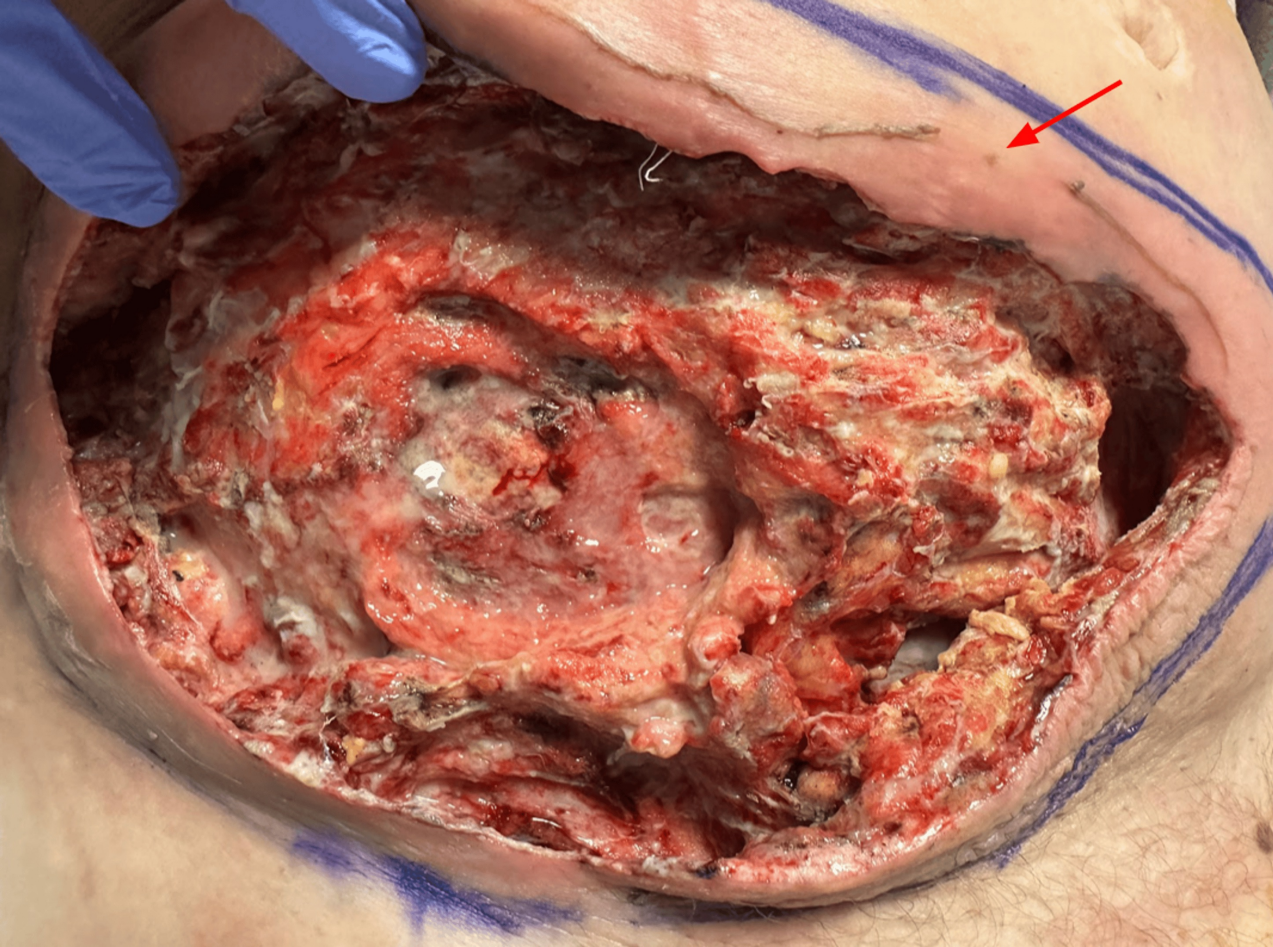
Postoperative image showing the anterior abdominal wall following wound debridement to viable tissue. There was no obvious violation of the abdominal fascia. Surrounding erythema borders marked.

**Figure 4:**
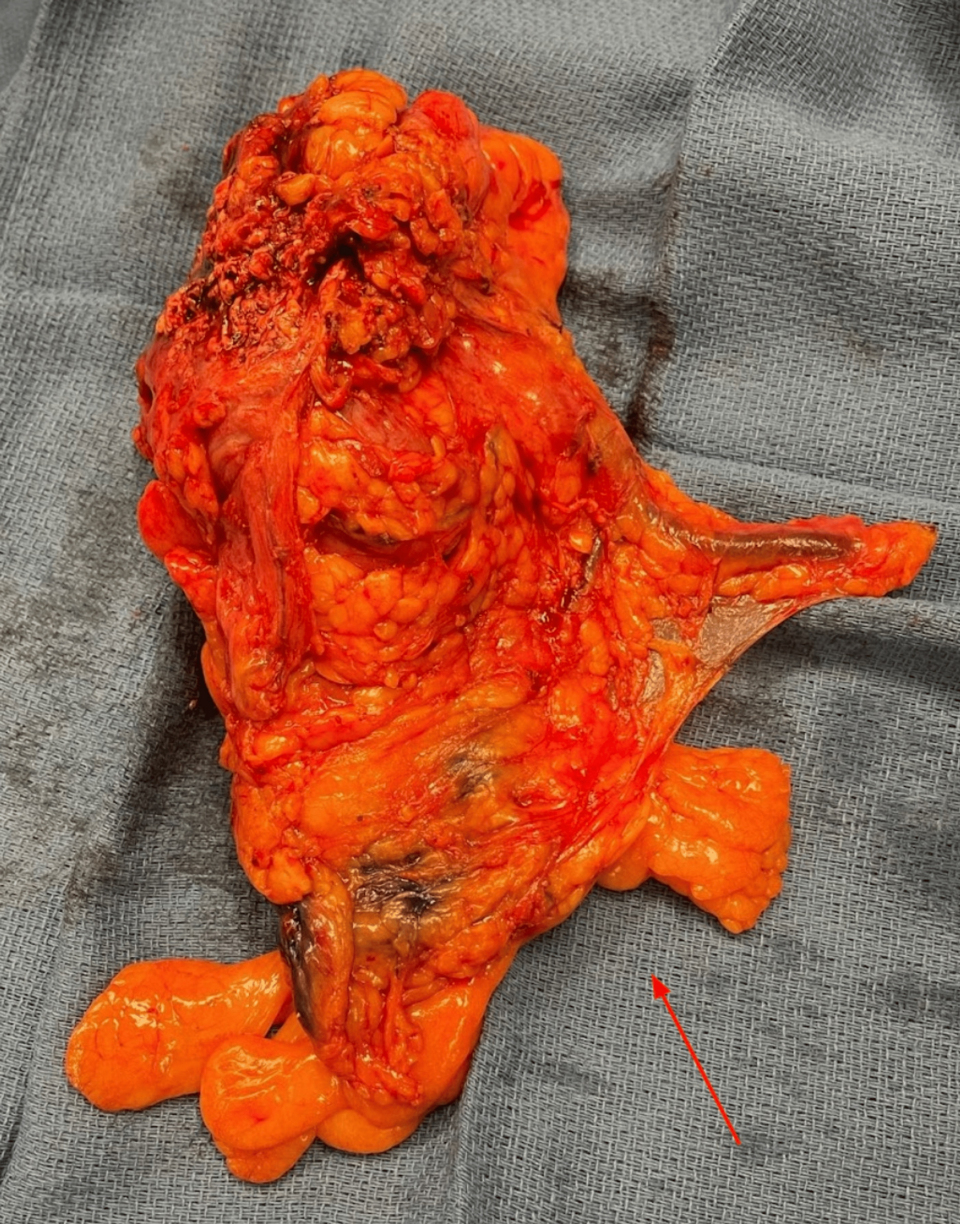
Excised specimen of right colon with evidence of a large cecal mass.

She was started on empiric antibiotics and transfused three units of packed red blood cells to achieve appropriate hematocrit levels. She then underwent bedside debridement of her abdominal wounds with eschar removal. However, due to the extensive disease burden, the decision was made to proceed with formal operative wound debridement. Intraoperatively, the wounds were debrided back to viable tissue with no obvious violation of abdominal fascia. Biopsies obtained resulted in marked active chronic inflammation and necrosis without evidence of malignancy. The two wounds were noted to be connected, and thus, the necrotic overlying skin was removed to create a single large wound, as seen in Figure 3. This was then packed with wet-to-dry gauze, and she was transferred to the surgical intensive care unit postoperatively. On postoperative Day 2, she underwent colonoscopy with findings notable for a large non-obstructing malignant appearing cecal mass. Biopsies tested positive for invasive adenocarcinoma. Following these findings, the decision was made to proceed with laparoscopic right colectomy with repeat wound debridement. Intraoperatively, the cecum was noted to be densely adhered to the anterior abdominal wall, surrounded by an inflammatory rind of necrotic material. A biopsy of the involved peritoneum was sent for pathologic analysis. The right colon was then mobilized up to the hepatic flexure, and extracorporeal resection with anastomosis was performed in standard fashion. The cecal mass within the excised specimen can be seen in Figure 4. Postoperatively, she recovered well without any significant complications. Final pathology revealed moderately differentiated adenocarcinoma with mucinous features and seven out of eleven positive lymph nodes, pathologic stage T3N2b. Notably, the peritoneal biopsy was negative for malignancy. She had a return of bowel function by postoperative Day 4 and was discharged home with intravenous antibiotics and negative pressure wound therapy. On outpatient follow-up two weeks later, she was noted to be recovering remarkably well with plans to start adjuvant chemotherapy one month later. 

## Discussion

The patient discussed above presented with rapidly progressive necrotic lower abdominal wounds and concern for systemic inflammatory response. This clinical presentation strongly supports the diagnosis of a necrotic soft tissue infection but offers no obvious indication of underlying malignancy, especially in this younger female. Local spread of colon cancer is common in advanced cases, showing direct invasion of nearby organs and possible peritoneal dissemination [4].

Studies have shown that only about 0.3-0.4% of cases perforate, causing intra-abdominal abscesses [3,4]. Furthermore, it is exceedingly rare for colon cancer to invade through the anterior abdominal wall to create a superficial abdominal wall abscess, and even more so to create a necrotizing infection. Unfortunately, these may be the first presenting symptoms [5-7]. In these rare presentations, challenges exist in both accurate diagnosis and definitive treatment.

The diagnosis of a necrotizing wound is a clinical one, but utilizing proper diagnostic imaging can be very useful if the diagnosis is uncertain or to provide information on the extent of the wound prior to invasive intervention [7,8]. In this case, CT imaging showed the extent of the wound while also hinting at concern for a large invasive cecal mass. This prompted endoscopic evaluation in a younger woman who was otherwise outside the recommended age for colon cancer screening. 

Interestingly, what grossly appeared to be an invasion of the tumor through the peritoneum intraoperatively, the peritoneal biopsy resulted negative for malignancy. While peritoneal biopsies have been shown to have a negative predictive value of up to 50% [9], it is more likely that the cecal tumor perforated, therefore facilitating local bacterial seeding into the abdominal wall to form a necrotizing infection. 

Management of the patient in our case presented a unique challenge due to balancing wound care and infection control with tumor resection and adjuvant therapy. Cases of abdominal wall abscesses secondary to penetrating colonic adenocarcinoma report using a variety of treatment methods, such as drainage of abscess followed by resection [5], preoperative radiation followed by resection [10], or a one-stage surgical resection of both the diseased colon and portion of the abdominal wall [3,6]. In this case, it was necessary to proceed with urgent wound debridement and subsequent removal of the tumor burden. Studies report that en bloc resection is the treatment of choice in the majority of cases; however, in certain circumstances, en bloc partial resection of the adherent abdominal wall may be performed secondary to inability to approximate a large defect and high risk of primary repair failure [4,7,11].

## Conclusions

This report presents a rare case of cecal adenocarcinoma presenting as a necrotizing wound infection. This case emphasizes the importance of a thorough history and physical, and to keep a high index of suspicion for underlying malignancy with a comprehensive workup to include early diagnostic imaging when atypical clinical scenarios such as this one present. It also emphasizes the need for individualized oncologic and wound care treatment to reduce morbidity and mortality, and thereby enhance overall patient outcomes.
